# Paliperidone palmitate vs. paliperidone extended-release for the acute treatment of adults with schizophrenia: a systematic review and pairwise and network meta-analysis

**DOI:** 10.1038/s41398-022-02286-1

**Published:** 2022-12-19

**Authors:** Taro Kishi, Kenji Sakuma, Nakao Iwata

**Affiliations:** Department of Psychiatry, Fujita Health University School of Medicine, Toyoake, Aichi Japan

**Keywords:** Schizophrenia, Drug discovery

## Abstract

Is paliperidone palmitate (PP) a useful treatment option for adults with acute symptoms of schizophrenia? We conducted a systematic review and a random-effects pairwise and network meta-analysis that compared PP (25−150 mg equivalent) with paliperidone extended-release (PAL-ER, 3−12 mg/d) regarding their efficacy and safety in adults with acute symptoms of schizophrenia. The outcomes were the total score of the Positive and Negative Syndrome Scale (PANSS-T) at week 6 (the primary outcome for efficacy) and all-cause discontinuation(the primary outcome for acceptability), discontinuation due to inefficacy, discontinuation due to adverse events, discontinuation due to the withdrawal of consent, and the incidence of individual adverse events. Five studies on PP and seven studies on PAL-ER, which involved 4970 individuals in total, were included in this study. For the primary outcomes, we only included data from the treatment arms that used 100 or 150 mg equivalent as an initial dose of PP and data from the treatment arms that used 6, 9, or 12 mg as an initial dose of PAL-ER. The pairwise meta-analyses showed that both PP and PAL-ER outperformed placebo regarding PANSS-T at week 6 and all-cause discontinuation. However, there were no statistically significant differences in these outcomes between the effect sizes of PP and that of PAL-ER. Both PP and PAL-ER increased blood prolactin levels in both females and males compared with placebo. PAL-ER significantly increased blood prolactin in both females and males compared with PP. There were no statistically significant differences in other outcomes between the effect sizes of PP and that of PAL-ER. Similar results in all outcomes were observed in the network meta-analyses. In conclusion, PP might be a useful treatment option for adults with acute symptoms of schizophrenia. A noninferiority study that directly compares PP with PAL-ER for acute schizophrenia, conducted according to the recommended regimen, is required to provide solid evidence.

## Introduction

Long-acting injection antipsychotics (LAI-APs) have been reported to reduce the risk of relapse in individuals with schizophrenia in the maintenance phase compared with oral antipsychotics (OAPs), according to a meta-analysis that included observational studies and a meta-analysis that included randomized controlled trials [1]. LAI-APs are considered to have some advantages over OAPs, such as improved pharmacokinetic profile (consistent bioavailability and more stable blood levels) and predictable adherence to the medication, all of which allow for lower dosages, though LAI-APs have disadvantages, such as injection site pain [2]. Drugs with less variation in peak and trough plasma concentrations (e.g., LAI-APs) may have fewer side effects and are more tolerable [3]. Because blood drug concentration gradually increases, most LAI-APs have been used for maintenance treatment rather than for acute treatment [2, 4].

Furthermore, we must address medication adherence issues for individuals with acute schizophrenia, as evidenced by the fact that even hospitalized individuals sometimes refuse to take medication. Antipsychotic medications must be administered to individuals with acute schizophrenia reliably and safely if their symptoms are to improve quickly. Short-acting injectable antipsychotics may be used for those who do not take OAPs in clinical practice [4]. Thus, we had the clinical question of whether LAI-APs, whose levels in the blood rise rapidly, are as effective as OAPs in treating adults with acute symptoms of schizophrenia or related disorders. In this study, we focused on paliperidone palmitate (PP). The recommended dose of PP is 150 mg equivalent on day 1 of treatment and 100 mg equivalent on day 8 [4]. Without the need for paliperidone extended-release (PAL-ER), this two-stage initiation regimen of PP allows for rapid therapeutically effective plasma concentrations [4]. Therefore, PP may be used to treat adults with acute symptoms of schizophrenia or related disorders similar to PAL-ER. To answer our clinical question, we conducted a systematic review and meta-analysis that compared PP with PAL-ER regarding their efficacy and safety in adults with acute symptoms of schizophrenia or related disorders.

## Methods

This systematic review and meta-analysis were conducted according to the Preferred Reporting Items for Systematic Reviews and Meta-Analyses statement (Table S1) [5, 6]. Two authors (TK and KS) worked simultaneously and independently on the literature search and data extraction, and the data obtained were input into a spreadsheet for analysis. The authors double-checked all data for accuracy. This study was registered at the Open Science Framework (https://osf.io/ykwzs).

### Search strategy and inclusion criteria

Figure S1 illustrates the formal literature search and selection flow of paliperidone trials. A formal systematic literature review was conducted using the Patient, Intervention, Comparison, Outcome strategy. Only double-blind, randomized controlled trials of PP or PAL-ER lasting at least 3 weeks were included. Studies including individuals with a dual diagnosis of the aforementioned mental illnesses and other disorders were excluded. Studies including children and adolescents were also excluded.

Patient: Adults with acute symptoms of schizophrenia spectrum and other psychotic disorders

Intervention: PP or PAL-ER

Comparison: Placebo (no trials comparing PP with PAL-ER have been conducted)

Outcomes: The total score of the Positive and Negative Syndrome Scale [7] (PANSS-T) at week 6 (primary outcome for efficacy), all-cause discontinuation (primary outcome for acceptability), discontinuation due to inefficacy, discontinuation due to adverse events, discontinuation due to the withdrawal of consent, and the incidence of individual adverse events. For studies in which the PANSS-T was displayed in a figure, it was measured from the curve using a ruler.

The authors searched for trials published before August 4, 2022 in the following databases: Embase, PubMed, and the Cochrane Central Register of Controlled Trials. We used the following search terms—(paliperidone) AND (schizophrenia) AND (random*)—in PubMed and the Cochrane Central Register of Controlled Trials databases. In Embase, we used the following search terms: (‘paliperidone’/exp OR paliperidone) AND (‘psychosis’/exp OR psychosis) AND (‘randomized controlled trial’/exp OR ‘randomized controlled trial’) in Embase. The literature search was performed without any language restriction. The authors evaluated the retrieved trials against the inclusion and exclusion criteria and selected those that were eligible. Additional relevant published and unpublished trials, including conference abstracts, were manually searched in the reference lists of the included trials and review articles. Clinical trial registries (e.g., ClinicalTrials.gov [http://clinicaltrials.gov/] and the World Health Organization International Clinical Trials Registry Platform [http://www.who.int/ictrp/search/en/]) were also searched to ensure that the eligible trials were comprehensive and to minimize the influence of publication bias. A consensus was achieved among the authors to resolve any discrepancy in the selected trials.

### Data synthesis and data extraction

The recommended dose of PP is 150 mg equivalent on day 1 of treatment and 100 mg equivalent on day 8 [4]. PP’s two-step initiation regimen enables the drug to be absorbed from two different sites, resulting in rapid therapeutically effective plasma concentrations without the need for oral supplementation [2]. The recommended maintenance dose was 25–150 mg equivalent/month [4]. Only one trial [8] followed the suggested protocol (Table S2). The peak plasma concentrations of PP are achieved at a median of 13 days after a single injection [9]. We only included data from the treatment arms that used 100 or 150 mg equivalent as an initial dose of PP in both the PANSS-T at week 6 and the rate of discontinuation because we considered that 25 and 50 mg equivalent were low as the dose for the initial and second injections, respectively, for these outcomes. For other outcomes, we included all approved doses of PP [4].

For PAL-ER, 6 mg/day is recommended as the initial dose. The recommended dose of PAL-ER was 3–12 mg/day. We considered that for PAL-ER, a fixed dose of 3 mg/day was low when evaluating the improvement of the outcomes related to the PANSS-T at week 6 and the rate of discontinuation. A dose of 15 mg/day was unapproved for PAL-ER. Therefore, data on 3 mg/day and 15 mg/day treatment arms were excluded from the outcomes related to the PANSS-T at week 6 and the rate of discontinuation in the PAL-ER group (Table S2). In the other outcomes, we included all approved doses of PAL-ER [4].

### Meta-analysis methods

Based on the aforementioned outcomes, a random-effects pairwise meta-analysis was performed to compare PP or PAL-ER with placebo [10]. We calculated the estimated mean differences (MD) in continuous data and risk ratios (RR) for dichotomous data, with their respective 95% confidence intervals (CIs). Then, subgroup differences were examined, comparing the effect size of individuals receiving PP with that of individuals receiving PAL-ER for each outcome. The heterogeneity of the included trials was evaluated using the *I*^2^ statistic, with *I*^2^ ≥ 50% considered as considerable heterogeneity [11]. Because we found considerable heterogeneity for the primary outcomes in the PAL-ER group, we performed a sensitivity pairwise analysis for the primary outcomes, excluding studies that only included individuals with schizoaffective disorder (Table S2) [12, 13]. Additionally, because the articles of the two trials did not mention whether the participants had acute symptoms [8, 14], we performed another sensitivity pairwise analysis for the primary outcomes, excluding these two trials. All pairwise meta-analyses were performed using Comprehensive Meta-Analysis, version 3 (Biostat Inc., Englewood, NJ, USA). We also used the random-effects model to perform frequentist network meta-analyses for all outcomes [15]. We used the τ^2^ statistic to assess network heterogeneity. The assumption of transitivity was tested by extracting potential effect modifiers, such as the mean PANSS-T at baseline, sample size, and mean age, and by comparing their distribution across comparisons in the network. We determined whether the distribution differences were large enough to threaten the validity of the analysis by comparing the distribution of these possible effect modifiers across the treatments included in the network meta-analysis using the Kruskal–Wallis test and by assessing their actual impact on the treatment effect through meta-regression analyses [16, 17]. We did not assess global statistical coherence and locally statistical coherence because due to a lack of data. Finally, we used the Confidence in Network Meta-Analysis (CINeMA) application to assess the credibility of the findings of each network meta-analysis, which is an adaptation of the Grading of Recommendations Assessment, Development, and Evaluation approach [18–20]. We evaluated the risk of bias using version 2 of the Cochrane risk-of-bias tool for randomized trials [11]. Because funnel plots with <10 studies were not meaningful [11], Egger’s test was used to determine potential publication bias.

## Results

### Study characteristics

The initial search retrieved 1331 articles, of which 497 were discarded as duplicates. Based on the review of the abstract and/or title of the remaining articles and trials, 811 were ruled out. The full text of the remaining 23 articles was reviewed, and 11 articles were excluded because they were post hoc studies (Fig. S1). Furthermore, the clinical trial registries revealed no further trials. Finally, five studies on PP [8, 14, 21–23] and seven studies on PAL-ER [12, 13, 24–28], involving 4,970 individuals in total, were included in this study. Only individuals with schizoaffective disorders were included in two of the trials [12, 13]. The articles of the two trials did not mention whether the participants had acute symptoms [8, 14]. However, because the mean PANSS-T at baseline was at least 80 for these two trials (Table S2), our meta-analysis included these trials. The participant inclusion criteria (Table S2), the total number of individuals, the proportion of males, and the mean age of the participants among all trials included in this meta-analysis were similar (Table S3). However, PAL-ER studies had a higher mean PANSS-T at baseline than PP studies (Table S3). Although all PAL-ER trials lasted 6 weeks, the duration of four of the five trials of PP was 13 weeks. All trials were industry-sponsored and were of high-quality design (Fig. S2).

### Pairwise meta-analysis

The pairwise meta-analysis showed that both PP and PAL-ER significantly decreased the PANSS-T at week 6 compared with placebo (Table 1). However, there was no significant difference in this efficacy outcome between effect size of PP and that of PAL-ER (Table 1). The result of the meta-analysis of PAL-ER in the primary efficacy outcome revealed significant heterogeneity (*I*^2^ = 69%). A sensitivity analysis excluding studies that only included individuals with schizoaffective disorder revealed that PAL-ER was also superior to placebo regarding the PANSS-T at week 6 (MD = −11.90, 95% CI = − 14.95, −8.85). However, considerable heterogeneity remained (*I*^2^ = 65%). Another sensitivity analysis excluding studies did not mention in their articles whether participants had acute symptoms revealed that PP was also superior to placebo in terms of the PANSS-T at week 6 (MD = − 9.09, 95% CI = − 13.52, −4.67, *I*^2^ = 49%). Egger’s test failed to detect statistically significant publication bias in both PP and PAL-ER meta-analyses of this outcome (data not shown).

**Table 1:** The result of the pairwise meta-analysis and the test for subgroup difference.

	Drug	*N*	Effect size (95% CI)^a^	*p* value	*I*^2^	Subgroup difference (*p* value)
PANSS total score at week 6	PAL-ER	7	−10.54 (−13.27, −7.81)	**<0.01**	69%	0.23
	PP	5	−8.08 (−11.01, −5.15)	**<0.01**	48%	
Discontinuation due to inefficacy	PAL-ER	7	0.48 (0.39, 0.60)	**<0.01**	20%	0.40
	PP	5	0.56 (0.44, 0.71)	**<0.01**	56%	
All-cause discontinuation	PAL-ER	7	0.67 (0.58, 0.77)	**<0.01**	62%	0.75
	PP	5	0.69 (0.59, 0.81)	**<0.01**	38%	
Discontinuation due to withdraw of consent	PAL-ER	7	1.05 (0.80, 1.38)	0.73	6%	0.95
	PP	5	0.10 (0.78, 1.38)	0.80	0%	
Discontinuation due to adverse events	PAL-ER	7	0.81 (0.56, 1.16)	0.25	0%	0.33
	PP	5	0.64 (0.46, 0.88)	**0.01**	18%	
Agitation	PAL-ER	5	0.75 (0.51, 1.12)	0.16	0%	0.60
	PP	4	0.88 (0.58, 1.34)	0.55	0%	
Use of anticholinergic agents	PAL-ER	7	1.39 (1.01, 1.92)	**0.046**	46%	0.41
	PP	4	1.13 (0.77, 1.66)	0.55	29%	
Akathisia	PAL-ER	6	1.12 (0.68, 1.84)	0.67	36%	0.97
	PP	3	1.10 (0.60, 2.00)	0.76	22%	
Extrapyramidal symptoms	PAL-ER	7	2.29 (1.48, 3.52)	**<0.01**	30%	0.29
	PP	4	1.55 (0.88, 2.74)	0.13	11%	
Somnolence	PAL-ER	6	1.46 (0.76, 2.84)	0.26	47%	0.66
	PP	3	1.97 (0.61, 6.32)	0.26	21%	
Dizziness	PAL-ER	7	1.22 (0.69, 2.16)	0.50	28%	0.78
	PP	4	1.40 (0.63, 3.12)	0.41	29%	
Insomnia	PAL-ER	7	0.93 (0.72, 1.21)	0.60	0%	0.81
	PP	4	0.89 (0.67, 1.18)	0.41	62%	
Headache	PAL-ER	6	1.02 (0.72, 1.47)	0.90	17%	0.61
	PP	4	1.21 (0.71, 2.07)	0.48	65%	
Constipation	PAL-ER	5	1.16 (0.60, 2.23)	0.66	60%	0.39
	PP	4	0.77 (0.41, 1.47)	0.43	3%	
Nausea	PAL-ER	4	0.85 (0.51, 1.40)	0.52	0%	0.73
	PP	4	0.98 (0.51, 1.87)	0.95	35%	
Vomiting	PAL-ER	4	0.89 (0.40, 1.98)	0.78	28%	0.61
	PP	4	1.18 (0.58, 2.43)	0.65	46%	
Weight gain	PAL-ER	6	1.90 (1.08, 3.37)	**0.03**	0%	0.56
	PP	5	2.36 (1.51, 3.69)	**<0.01**	0%	
Weight change (kg)	PAL-ER	7	1.16 (0.71, 1.60)	**<0.01**	64%	0.12
	PP	4	1.80 (1.14, 2.45)	**<0.01**	49%	
Blood prolactin change in male (ng/mL)	PAL-ER	6	21.99 (18.55, 25.44)	**<0.01**	65%	**<0.01**
	PP	4	10.76 (7.05, 14.46)	**<0.01**	11%	
Blood prolactin change in female (ng/mL)	PAL-ER	6	76.34 (62.97, 89.71)	**<0.01**	27%	**<0.01**
	PP	4	45.29 (34.01, 56.57)	**<0.01**	48%	

Bold values denote statistical significance at the p < 0.05 level.

*95% CI* 95% confidence interval, *PAL-ER* paliperidone extended-release, *PANSS* Positive and Negative Syndrome Scale, *PP* paliperidone palmitate.

^a^The underlined effect size was the mean difference. Other effect sizes were risk ratios.

The pairwise meta-analysis showed that both PP and PAL-ER were associated with lower all-cause discontinuation than placebo (Table 1). However, there was no statistically significant difference in the outcome between the effect size of PP and that of PAL-ER (Table 1). The result of the meta-analysis of PAL-ER showed considerable heterogeneity (*I*^2^ = 62%). A sensitivity analysis that excluded studies that included individuals with schizoaffective disorder revealed that PAL-ER was also superior to placebo in terms of all-cause discontinuation (RR = 0.64, 95% CI = 0.54, 0.76). However, considerable heterogeneity remained (*I*^2^ = 69%). Another sensitivity analysis excluding studies did not mention in their articles whether participants had acute symptoms revealed that PP was also superior to placebo in terms of all-cause discontinuation (RR = 0.74, 95% CI = 0.56, 0.88, *I*^2^ = 42%). Egger’s test failed to detect statistically significant publication bias for both meta-analyses of PP and PAL-ER regarding this outcome (data not shown).

The pairwise meta-analysis showed that both PP and PAL-ER were associated with a lower rate of discontinuation due to inefficacy than placebo (Table 1). Although neither treatment outperformed placebo in terms of the rate of discontinuation due to withdrawal of consent, only PP was associated with a lower rate of discontinuation due to adverse events than placebo (Table 1). However, no significant differences in these outcomes were found between the effect sizes of PP and that of PAL-ER (Table 1).

The pairwise meta-analysis revealed that only PAL-ER was associated with a higher incidence of anticholinergic agent use and extrapyramidal symptoms than placebo (Table 1). PP and PAL-ER were associated with a higher incidence of weight gain and increased body weight than placebo (Table 1). However, no significant differences in these outcomes were found between the effect sizes of PP and that of PAL-ER (Table 1). Both PP and PAL-ER increased blood prolactin levels in both females and males compared with placebo (Table 1). PAL-ER significantly increased blood prolactin in both females and males compared with PP (Table 1). Other adverse events were not significantly different between the PP or PAL-ER group and the placebo group (Table 1).

### Network meta-analysis

**Fig. 1:**
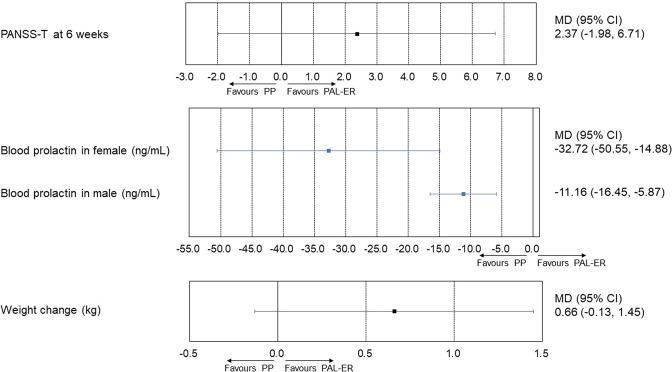
The results of the network meta-analysis: continuous variables. Error bar represents 95% CI. PP was compared with PAL-ER. Colors indicate the presence or absence of a significant difference: blue, PP was superior to PAL-ER; black, PP was similar to PAL-ER. 95% CI, 95% confidence interval; MD, mean difference; PAL-ER, paliperidone extended-release; PANSS-T, total score of the Positive and Negative Syndrome Scale total score; PP, paliperidone palmitate.

Because there was not a direct comparison of PP with PAL-ER for acute schizophrenia (Table S2), the results of the network meta-analysis comparing PP with PAL-ER for all outcomes were only indirect evidence. Although PAL-ER significantly increased blood prolactin in both females and males compared with PP (Fig. 1), no significant differences in other outcomes were observed between PP and PAL-ER, according to the network meta-analysis (Fig. 1 and Fig. S3). Our meta-regression analyses showed no association between the mean PANSS-T at baseline and the effect sizes of the primary outcomes (coefficient [95% CI]: PANSS-T at week 6 = −1.77 [−9.39, 6.53], all-cause discontinuation = 0.10 [−0.33, 0.47]). In most outcomes, global heterogeneity was evaluated as low (Table S4). However, in the network meta-analysis, the confidence rating for indirect comparison was reduced to one level. Furthermore, because a test for publication bias with <10 studies was ineffective [11], CINeMA rated all comparisons for publication bias as “some concerns.” Consequently, the network meta-analysis showed a low level of confidence in all outcomes (Table S4).

## Discussion

In this meta-analysis, we discovered that PP and PAL-ER might be equally effective in treating adults with acute schizophrenia. In contrast, PP had a significantly lower risk of increased blood prolactin than PAL-ER, despite both having a risk of increased blood prolactin. This meta-analysis found that PAL-ER, but not PP, had a higher risk of extrapyramidal symptom-related adverse events than placebo. PP was well-tolerated by adults with acute symptoms of schizophrenia or related disorders because it was associated with a lower rate of discontinuation due to adverse events than placebo. Based on our findings, PP might be a useful treatment option for adults with acute symptoms of schizophrenia or related disorders. Recent evidence‑based expert consensus recommends that LAI-APs could be initiated both during an acute psychotic episode and when patients are stable [29]. The results of this study on PP support this recommendation. However, a noninferiority study that directly compares PP with PAL-ER for acute schizophrenia, conducted according to the recommended regimen, is required to provide solid evidence.

This study had several limitations. First, the numbers of studies and patients were small. Second, the duration of the trials included in this study was short. Third, our meta-analysis included only double-blind randomized controlled trials, and thus, homogeneous patient cohorts and strictly controlled clinical environments associated with randomized controlled trials may not reflect real-world practice and outcomes [30]; therefore, our results might have reflected selection bias. However, to date, no large cohort studies in which PP was compared with PAL-ER for acute schizophrenia have been reported. Fourth, because there was of direct comparison of PP with PAL-ER for acute schizophrenia, the results of the network meta-analysis comparing PP with PAL-ER for all outcomes were only indirect evidence. Fifth, the articles of two trials did not mention whether participants had acute symptoms [8, 14]. However, our sensitivity pairwise meta-analyses that excluded studies that did not mention in their articles whether participants had acute symptoms had results for the primary outcomes similar to those of the original pairwise meta-analyses. Additionally, because the minimum PANSS-T at baseline in all trials included in our meta-analysis was relatively low (60 or 70)(Table S2), the PANSS-T at baseline among those trials might be skewed. We found a significant difference in the mean PANSS-T at baseline between PP and PAL-ER studies (Table S3). However, our meta-regression analyses did not show an association between the mean PANSS-T at baseline and the effect size of the primary outcomes. Sixth, although the number of studies on other LAI-APs, such as aripiprazole, for adults with acute symptoms of schizophrenia was insufficient to conduct a meta-analysis at this time, these LAI-APs should be investigated in the future. Finally, we did not cover important clinical issues that might inform treatment decision-making in routine clinical practice (e.g., cost-effectiveness and combination with nonpharmacological treatments).

In conclusion, PP might be a useful treatment option for adults with acute symptoms of schizophrenia or related disorders. A noninferiority study that directly compares PP with PAL-ER for acute schizophrenia, conducted according to the recommended regimen, is required to provide solid evidence.

## Supplementary information

Supplementary materials

## Data Availability

Data used for the current study were reported in articles as cited in this paper.
