# Supplementary material for: Paliperidone palmitate vs. paliperidone extended-release for the acute treatment of adults with schizophrenia: a systematic review and pairwise and network meta-analysis
Source: Transl Psychiatry. 2022 Dec 19;12:519. doi: 10.1038/s41398-022-02286-1 (PMC9763417; doi:10.1038/s41398-022-02286-1)
Supplement: Supplementary file 1 — Supplementary materials [file 41398_2022_2286_MOESM1_ESM.pdf]

Figure S1. Flow diagram of literature search.

The initial search retrieved 1331 articles, of which 497 were discarded as duplicates. Based on the review of the abstract and/or title of the remaining articles and trials, 811 were ruled out. The full text of the remaining 23 articles was reviewed, and eleven articles were excluded because they were post-hoc studies. Furthermore, the clinical trial registries revealed no further trials. Finally, five studies of PP (Gopal et al. 2010; Kramer et al. 2010; Nasrallah et al. 2010; Pandina et al. 2010; Takahashi et al. 2013) and seven studies of OP (Canuso et al. 2010a; Canuso et al. 2010b; Coppola et al. 2011; Davidson et al. 2007; Hirayasu et al. 2010; Kane et al. 2007; Marder et al. 2007) were included in the current study (total n=4,970).

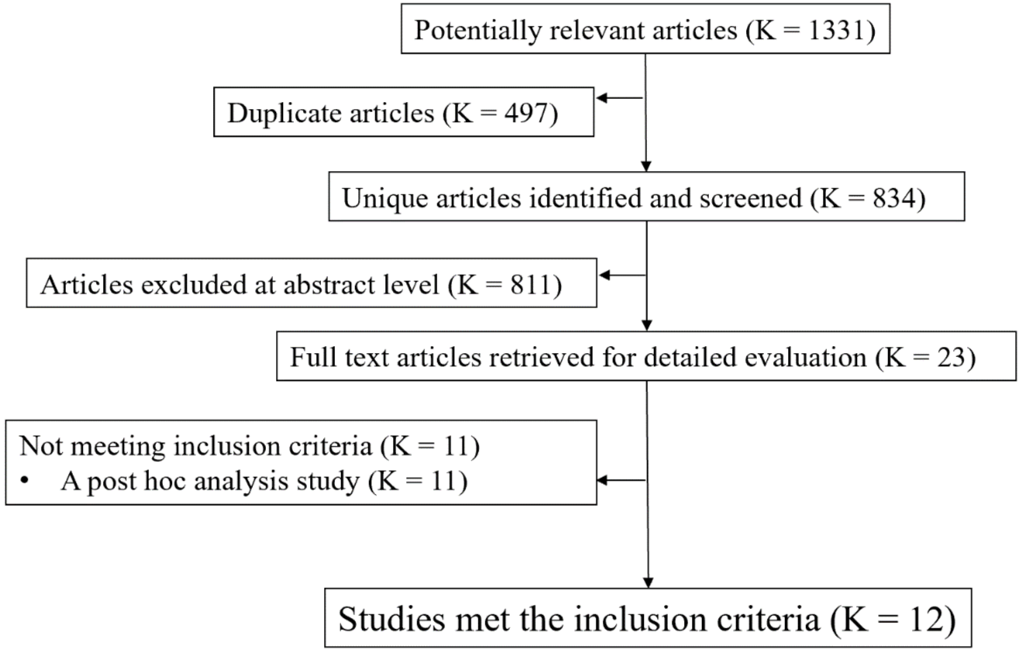

Review articles used in the hand search (4 articles)

Huhn M, Nikolakopoulou A, Schneider-Thoma J, Krause M, Samara M, Peter N, Arndt T, Backers L, Rothe P, Cipriani A, Davis J, Salanti G, Leucht S (2019) Comparative efficacy and tolerability of 32 oral antipsychotics for the acute treatment of adults with multi-episode schizophrenia: a systematic review and network meta-analysis. Lancet 394: 939-951.

Kishi T, Ikuta T, Sakuma K, Okuya M, Iwata N (2021) Efficacy and safety of antipsychotic treatments for schizophrenia: A systematic review and network meta-analysis of randomized trials in Japan. J Psychiatr Res 138: 444-452.

Nussbaum A, Stroup TS (2008) Paliperidone for schizophrenia. Cochrane Database Syst Rev: CD006369.

Nussbaum AM, Stroup TS (2012) Paliperidone palmitate for schizophrenia. Cochrane Database Syst Rev: CD008296.

Figure S2. Risk of bias summary.

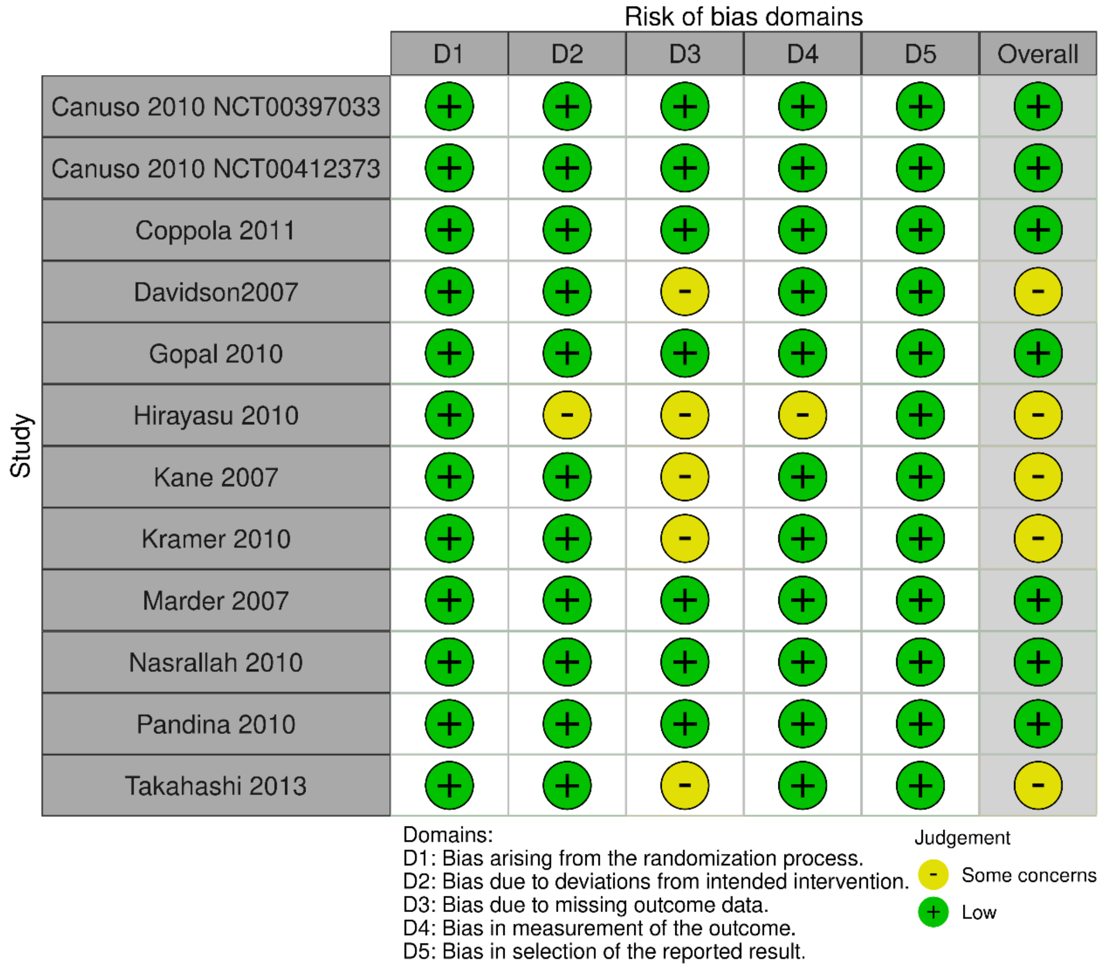

We referred to the following article:

Schneider-Thoma J, et al., Comparative efficacy and tolerability of 32 oral and long-acting injectable antipsychotics for the maintenance treatment of adults with schizophrenia: a systematic review and network meta-analysis. Lancet. 2022 Feb 26;399(10327):824-836.

Figure S3. The results of the network meta-analysis: dichotomous variables.

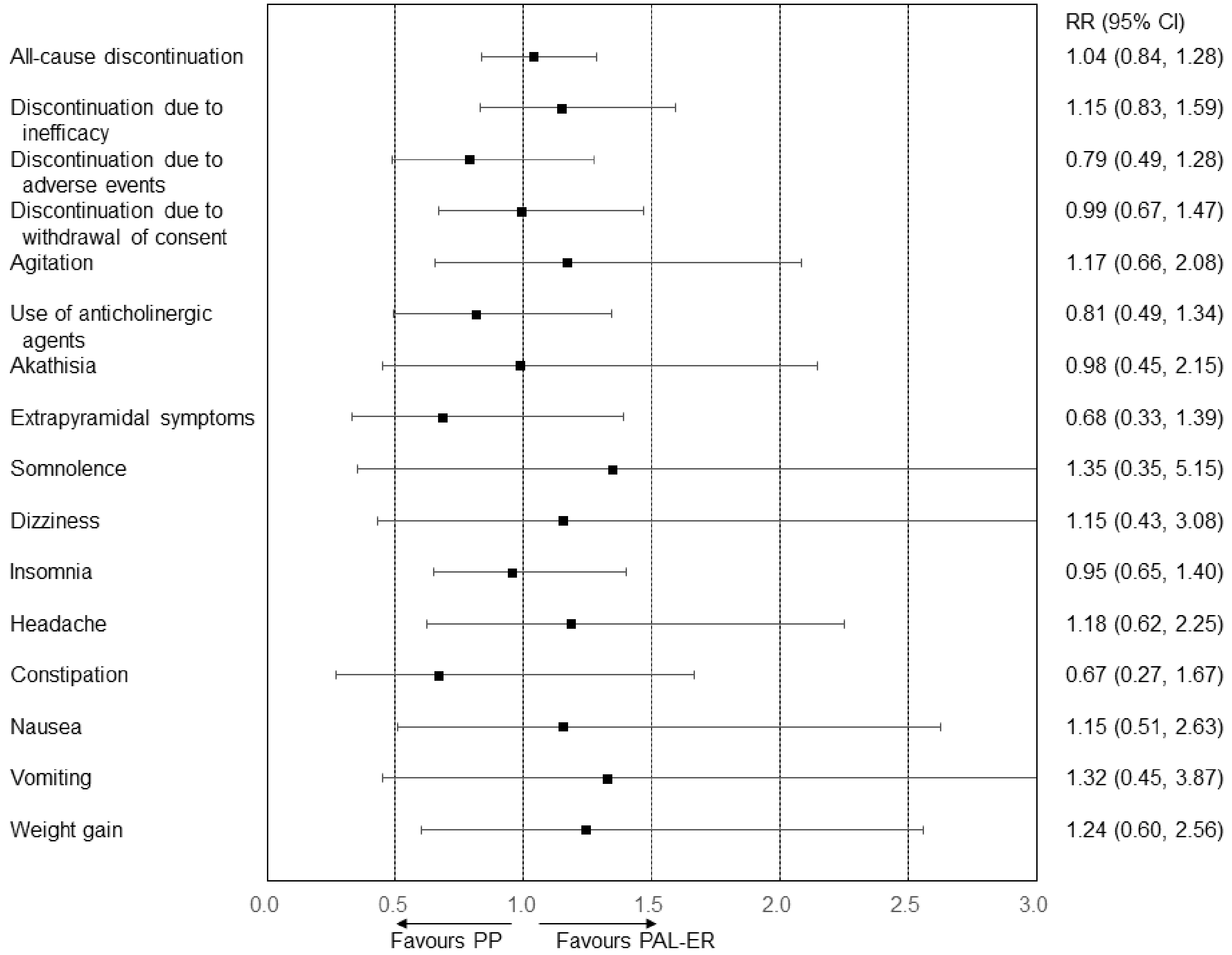

Error bar represents 95% CI

95% CI: 95% confidence interval, PAL-ER: paliperidone extended-release, PP: paliperidone palmitate, RR: risk ratio

Table S1.1. PRISMA for Pairwise Meta-Analyses Checklist.

Section and Topic	Item #	Checklist item	Location where item is reported
**TITLE**			
Title	1	Identify the report as a systematic review.	1
**ABSTRACT**			
Abstract	2	See the PRISMA 2020 for Abstracts checklist.	none
**INTRODUCTION**			
Rationale	3	Describe the rationale for the review in the context of existing knowledge.	2-3
Objectives	4	Provide an explicit statement of the objective(s) or question(s) the review addresses.	2-3
**METHODS**			
Eligibility criteria	5	Specify the inclusion and exclusion criteria for the review and how studies were grouped for the syntheses.	3-4
Information sources	6	Specify all databases, registers, websites, organisations, reference lists and other sources searched or consulted to identify studies. Specify the date when each source was last searched or consulted.	3-4
Search strategy	7	Present the full search strategies for all databases, registers and websites, including any filters and limits used.	3-4
Selection process	8	Specify the methods used to decide whether a study met the inclusion criteria of the review, including how many reviewers screened each record and each report retrieved, whether they worked independently, and if applicable, details of automation tools used in the process.	3-4
Data collection process	9	Specify the methods used to collect data from reports, including how many reviewers collected data from each report, whether they worked independently, any processes for obtaining or confirming data from study investigators, and if applicable, details of automation tools used in the process.	3-4
Data items	10a	List and define all outcomes for which data were sought. Specify whether all results that were compatible with each outcome domain in each study were sought (e.g. for all measures, time points, analyses), and if not, the methods used to decide which results to collect.	3-4
	10b	List and define all other variables for which data were sought (e.g. participant and intervention characteristics, funding sources). Describe any assumptions	3-4

Section and Topic	Item #	Checklist item	Location where item is reported
		made about any missing or unclear information.	
Study risk of bias assessment	11	Specify the methods used to assess risk of bias in the included studies, including details of the tool(s) used, how many reviewers assessed each study and whether they worked independently, and if applicable, details of automation tools used in the process.	3-4
Effect measures	12	Specify for each outcome the effect measure(s) (e.g. risk ratio, mean difference) used in the synthesis or presentation of results.	3-4
Synthesis methods	13a	Describe the processes used to decide which studies were eligible for each synthesis (e.g. tabulating the study intervention characteristics and comparing against the planned groups for each synthesis (item #5)).	3-4
	13b	Describe any methods required to prepare the data for presentation or synthesis, such as handling of missing summary statistics, or data conversions.	3-4
	13c	Describe any methods used to tabulate or visually display results of individual studies and syntheses.	3-4
	13d	Describe any methods used to synthesize results and provide a rationale for the choice(s). If meta-analysis was performed, describe the model(s), method(s) to identify the presence and extent of statistical heterogeneity, and software package(s) used.	3-4
	13e	Describe any methods used to explore possible causes of heterogeneity among study results (e.g. subgroup analysis, meta-regression).	3-4
	13f	Describe any sensitivity analyses conducted to assess robustness of the synthesized results.	3-4
Reporting bias assessment	14	Describe any methods used to assess risk of bias due to missing results in a synthesis (arising from reporting biases).	3-4
Certainty assessment	15	Describe any methods used to assess certainty (or confidence) in the body of evidence for an outcome.	3-4
**RESULTS**			
Study selection	16a	Describe the results of the search and selection process, from the number of records identified in the search to the number of studies included in the review, ideally using a flow diagram.	4-6

Section and Topic	Item #	Checklist item	Location where item is reported
	16b	Cite studies that might appear to meet the inclusion criteria, but which were excluded, and explain why they were excluded.	
Study characteristics	17	Cite each included study and present its characteristics.	4-6
Risk of bias in studies	18	Present assessments of risk of bias for each included study.	4-6
Results of individual studies	19	For all outcomes, present, for each study: (a) summary statistics for each group (where appropriate) and (b) an effect estimate and its precision (e.g. confidence/credible interval), ideally using structured tables or plots.	4-6
Results of syntheses	20a	For each synthesis, briefly summarise the characteristics and risk of bias among contributing studies.	4-6
	20b	Present results of all statistical syntheses conducted. If meta-analysis was done, present for each the summary estimate and its precision (e.g. confidence/credible interval) and measures of statistical heterogeneity. If comparing groups, describe the direction of the effect.	4-6
	20c	Present results of all investigations of possible causes of heterogeneity among study results.	4-6
	20d	Present results of all sensitivity analyses conducted to assess the robustness of the synthesized results.	4-6
Reporting biases	21	Present assessments of risk of bias due to missing results (arising from reporting biases) for each synthesis assessed.	4-6
Certainty of evidence	22	Present assessments of certainty (or confidence) in the body of evidence for each outcome assessed.	4-6
**DISCUSSION**			
Discussion	23a	Provide a general interpretation of the results in the context of other evidence.	6-7
	23b	Discuss any limitations of the evidence included in the review.	6-7
	23c	Discuss any limitations of the review processes used.	6-7

Section and Topic	Item #	Checklist item	Location where item is reported
	23d	Discuss implications of the results for practice, policy, and future research.	
**OTHER INFORMATION**			
Registration and protocol	24a	Provide registration information for the review, including register name and registration number, or state that the review was not registered.	3
	24b	Indicate where the review protocol can be accessed, or state that a protocol was not prepared.	3
	24c	Describe and explain any amendments to information provided at registration or in the protocol.	3
Support	25	Describe sources of financial or non-financial support for the review, and the role of the funders or sponsors in the review.	7
Competing interests	26	Declare any competing interests of review authors.	7-8
Availability of data, code and other materials	27	Report which of the following are publicly available and where they can be found: template data collection forms; data extracted from included studies; data used for all analyses; analytic code; any other materials used in the review.	8

Table S1.2. PRISMA for Network Meta-Analyses Checklist.

Section/Topic	Item #	Checklist Item	Reported on Page #
**TITLE**			
Title	1	Identify the report as a systematic review *incorporating a network meta-analysis (or related form of meta-analysis).*	1
**ABSTRACT**			
Structured summary	2	Provide a structured summary including, as applicable:<br>**Background:** main objectives<br>**Methods:** data sources; study eligibility criteria, participants, and interventions; study appraisal; and *synthesis methods, such as network meta-analysis.*<br>**Results:** number of studies and participants identified; summary estimates with corresponding confidence/credible intervals; *treatment rankings may also be discussed. Authors may choose to summarize pairwise comparisons against a chosen treatment included in their analyses for brevity.*<br>**Discussion/Conclusions:** limitations; conclusions and implications of findings.<br>**Other:** primary source of funding; systematic review registration number with registry name.	none
**INTRODUCTION**			
Rationale	3	Describe the rationale for the review in the context of what is already known, *including mention of why a network meta-analysis has been conducted.*_	2-3
Objectives	4	Provide an explicit statement of questions being addressed, with reference to participants, interventions, comparisons, outcomes, and study design (PICOS).	2-3
**METHODS**			

Protocol and registration	5	Indicate whether a review protocol exists and if and where it can be accessed (e.g., Web address); and, if available, provide registration information, including registration number.	3-4
Eligibility criteria	6	Specify study characteristics (e.g., PICOS, length of follow-up) and report characteristics (e.g., years considered, language, publication status) used as criteria for eligibility, giving rationale. *Clearly describe eligible treatments included in the treatment network, and note whether any have been clustered or merged into the same node (with justification).*_	3-4
Information sources	7	Describe all information sources (e.g., databases with dates of coverage, contact with study authors to identify additional studies) in the search and date last searched.	3-4
Search	8	Present full electronic search strategy for at least one database, including any limits used, such that it could be repeated.	3-4
Study selection	9	State the process for selecting studies (i.e., screening, eligibility, included in systematic review, and, if applicable, included in the meta-analysis).	3-4
Data collection process	10	Describe method of data extraction from reports (e.g., piloted forms, independently, in duplicate) and any processes for obtaining and confirming data from investigators.	3-4
Data items	11	List and define all variables for which data were sought (e.g., PICOS, funding sources) and any assumptions and simplifications made.	3-4
**Geometry of the network**	**S1**	Describe methods used to explore the geometry of the treatment network under study and potential biases related to it. This should include how the evidence base has been graphically summarized for presentation, and what characteristics were compiled and used to describe the evidence base to readers.	3-4
Risk of bias within individual studies	12	Describe methods used for assessing risk of bias of individual studies (including specification of whether this was done at the study or outcome level), and how this information is to be used in any data synthesis.	3-4
Summary measures	13	State the principal summary measures (e.g., risk ratio, difference in means). *Also describe the use of additional summary measures assessed, such as treatment rankings and surface under the cumulative ranking curve (SUCRA)* values, as well as modified approaches used to present summary findings from meta-analyses.*	3-4

Planned methods of analysis	14	Describe the methods of handling data and combining results of studies for each network meta-analysis. This should include, but not be limited to:<br>• *Handling of multi-arm trials;*<br>• *Selection of variance structure;*<br>• *Selection of prior distributions in Bayesian analyses; and*<br>• *Assessment of model fit.*	3-4
**Assessment of Inconsistency**	**S2**	Describe the statistical methods used to evaluate the agreement of direct and indirect evidence in the treatment network(s) studied. Describe efforts taken to address its presence when found.	3-4
Risk of bias across studies	15	Specify any assessment of risk of bias that may affect the cumulative evidence (e.g., publication bias, selective reporting within studies).	3-4
Additional analyses	16	Describe methods of additional analyses if done, indicating which were pre-specified. This may include, but not be limited to, the following:<br>• Sensitivity or subgroup analyses;<br>• Meta-regression analyses;<br>• *Alternative formulations of the treatment network; and*<br>• *Use of alternative prior distributions for Bayesian analyses (if applicable).*_	3-4
**RESULTS†**			
Study selection	17	Give numbers of studies screened, assessed for eligibility, and included in the review, with reasons for exclusions at each stage, ideally with a flow diagram.	4-6
**Presentation of network structure**	**S3**	Provide a network graph of the included studies to enable visualization of the geometry of the treatment network.	4-6
**Summary of network geometry**	**S4**	Provide a brief overview of characteristics of the treatment network. This may include commentary on the abundance of trials and randomized patients for the different interventions and pairwise comparisons in the network, gaps of evidence in the treatment network, and potential biases reflected	4-6

		by the network structure.	
Study characteristics	18	For each study, present characteristics for which data were extracted (e.g., study size, PICOS, follow-up period) and provide the citations.	4-6
Risk of bias within studies	19	Present data on risk of bias of each study and, if available, any outcome level assessment.	4-6
Results of individual studies	20	For all outcomes considered (benefits or harms), present, for each study: 1) simple summary data for each intervention group, and 2) effect estimates and confidence intervals. *Modified approaches may be needed to deal with information from larger networks.*	4-6
Synthesis of results	21	Present results of each meta-analysis done, including confidence/credible intervals. *In larger networks, authors may focus on comparisons versus a particular comparator (e.g. placebo or standard care), with full findings presented in an appendix. League tables and forest plots may be considered to summarize pairwise comparisons.* If additional summary measures were explored (such as treatment rankings), these should also be presented.	4-6
**Exploration for inconsistency**	**S5**	Describe results from investigations of inconsistency. This may include such information as measures of model fit to compare consistency and inconsistency models, *P* values from statistical tests, or summary of inconsistency estimates from different parts of the treatment network.	4-6
Risk of bias across studies	22	Present results of any assessment of risk of bias across studies for the evidence base being studied.	4-6
Results of additional analyses	23	Give results of additional analyses, if done (e.g., sensitivity or subgroup analyses, meta-regression analyses, *alternative network geometries studied, alternative choice of prior distributions for Bayesian analyses*, and so forth).	4-6
**DISCUSSION**			
Summary of evidence	24	Summarize the main findings, including the strength of evidence for each main outcome; consider their relevance to key groups (e.g., healthcare providers, users, and policy-makers).	6-7

Limitations	25	Discuss limitations at study and outcome level (e.g., risk of bias), and at review level (e.g., incomplete retrieval of identified research, reporting bias). *Comment on the validity of the assumptions, such as transitivity and consistency. Comment on any concerns regarding network geometry (e.g., avoidance of certain comparisons).*	6-7
Conclusions	26	Provide a general interpretation of the results in the context of other evidence, and implications for future research.	6-7
**FUNDING**			7
Funding	27	Describe sources of funding for the systematic review and other support (e.g., supply of data); role of funders for the systematic review. This should also include information regarding whether funding has been received from manufacturers of treatments in the network and/or whether some of the authors are content experts with professional conflicts of interest that could affect use of treatments in the network.	

* SUCRA values were not calculated because only three treatment conditions were used.

Table S2. Included double-blind, randomized placebo-controlled trials in the current systematic review.

(1) Study name*<br>(2) Trial ID	(1) Total n<br>(2) Study duration	(1) Diagnosis<br>(2) Characteristics of the patient	Treatment*****		Proportion of male (%)	Mean age (years)	Mean PANSS-T at baseline
(1) Canuso 2010<br>(2) NCT00397033	(1) 316<br>(2) 6 weeks	(1) IP with SA (DSM-IV)<br>(2) PANSS-T≥60, YMRS and/or HAMD21≥16	PAL-ER 3-6 mg/day (starting dose 6 mg/day)		66.7	38.1±10.0	95.9±13.0
			PAL-ER 9-12 mg/day (starting dose 12 mg/day)		65.3	36.5±10.5	92.7±12.6
			Placebo		62.6	37.1±11.1	91.6±12.5
(1) Canuso 2010<br>(2) NCT00412373	(1) 311<br>(2) 6 weeks	(1) IP with SA (DSM-IV)<br>(2) PANSS-T≥60, YMRS and/or HAMD21≥16	PAL-ER 3-12 mg/day (starting dose 6 mg/day)		55.0	37.7±9.1	92.3±13.5
			Placebo		58.1	37.3±9.4	91.7±12.1
(1) Coppola 2011**<br>(2) NCT00524043	(1) 201<br>(2) 6 weeks	(1) IP with SZ or SA (DSM-IV)<br>(2) PANSS-T=70-120	PAL-ER 6 mg/day		69.5	40.7±12.2	92.6±13.0
			Placebo		73.6	36.4±10.7	
(1) Davidson 2007***<br>(2) NCT00083668	(1) 618<br>(2) 6 weeks	(1) IP with SZ (DSM-IV)<br>(2) PANSS-T=70-120	PAL-ER 3 mg/day		63	36.3±11.0	91.6±12.2
			PAL-ER 9 mg/day		64	36.2±10.9	93.9±13.2
			Placebo		69	37.3±10.9	93.9±12.7
(1) Gopal 2010****<br>(2) NCT00147173	(1) 388<br>(2) 13 weeks	(1) IP with SZ (DSM-IV)<br>(2) PANSS-T=70-120, BMI>17	PP1M 50 mg eq.	The first 2 injections on days 1 and 8 and the next 2 injections on days 36 and 64.	69.9	39±10.6	90±10.8
			PP1M 100 mg eq.		64.9	39±10.7	90±11.7
			PP1M 150 mg eq.		73.3	41±11.1	92±11.7
			Placebo		71.2	41±11.0	92±12.6
(1) Hirayasu 2010 (Japan)<br>(2) NCT00396565	(1) 321<br>(2) 6 weeks	(1) IP with SZ (DSM-IV-TR)<br>(2) PANSS-T=70-120	PAL-ER 6 mg/day		52.2	44.0±14.7	92.0±12.9
			Placebo		52.2	46.2±15.1	91.0±11.2
(1) Kane 2007<br>(2) NCT00078039	(1) 630<br>(2) 6 weeks	(1) IP with SZ (DSM-IV)<br>(2) PANSS-T=70-120	PAL-ER 6 mg/day		50	37.0±10.2	94.3±10.5
			PAL-ER 9 mg/day		59	38.5±11.4	93.2±11.9
			PAL-ER 12 mg/day		53	36.0±10.6	94.6±11.0
			Placebo		52	37.9±10.9	94.1±10.7

(1) Kramer 2010	(1) 247	(1) IP with SZ (DSM-IV)	PP1M 50 mg eq.	Preceding 7 days oral PAL phase. The first 2 injections on days 1 and 8 and the next injection on day 36.	65.1	40±9.8	88.0±12.4
(2) NCT00074477	(2) 9 weeks	(2) PANSS-T=70-120, BMI=15-35	PP1M 100 mg eq.		61.8	37±10.4	85.2±11.1
			Placebo		59.1	40±10.5	87.8±13.9
(1) Marder 2007 (USA)	(1) 444	(1) IP with SZ (DSM-IV)	PAL-ER 6 mg/day		74	41.6	92.3±12.0
(2) NCT00077714	(2) 6 weeks	(2) PANSS-T=70-120	PAL-ER 12 mg/day				94.1±11.4
			Placebo				93.6±11.7
(1) Nasrallah 2010	(1) 518	(1) IP with SZ (DSM-IV-TR)	PP1M 25 mg eq.	The first 2 injections on days 1 and 8 and the next 2 injections on days 36 and 64.	65	40.8±10.6	90.6±12.2
(2) NCT00101634	(2) 13 weeks	(2) PANSS-T=70-120, BMI>15	PP1M 50 mg eq.		73	39.0±11.9	91.2±12.0
			PP1M 100 mg eq.		65	42.3±10.7	90.8±11.7
			Placebo		62	41.1±11.8	90.7±12.2
(1) Pandina 2010	(1) 652	(1) IP with SZ (DSM-IV)	PP1M 25 mg eq.	PP 150 mg eq. on day 1, and the assigned treatment on days 8, 36, and 64.	72.5	39.2±10.3	87.1
(2) NCT00590577	(2) 13 weeks	(2) PANSS-T=70-120, BMI<40	PP1M 100 mg eq.		66.7	38.7±10.3	
			PP1M 150 mg eq.		64.4	39.4±10.7	
			Placebo		66.5	39.9±11.0	
(1) Takahashi 2013	(1) 324	(1) IP with SZ (DSM-IV-TR)	PP1M 75-150 mg eq.	PP 150 mg eq. on day 1, PP 100 mg eq. on day 8, and PP 75 mg. eq on days 36 and 64.	63.5	46±13.6	85.7±14.6
(2) NCT01299389	(2) 13 weeks	(2) PANSS-T=60-120	Placebo		50.6	44±12.4	83.5±15.2

IP: inpatient, SA: schizoaffective disorder, SZ: schizophrenia
* The underlined studies were conducted in several countries.
**PAL-ER 1.5 mg/d arm was not included in our study because it was not recommended dose
***PAL-ER 15 mg/d arm was not included in our study because it was not recommended dose
****PP 150 mg eq./placebo arm was not included in our study because the arm had a medication allocation error according to our study.
*****PP doses are measured in milligram or milligram equivalents (mg eq) of active paliperidone (39, 78, 117, 156, and 234 mg of PP correspond to 25, 50, 75, 100, and 150 mg eq of paliperidone).

Adults with acute symptoms of schizophrenia or related disorders were included in all trials. Only individuals with schizoaffective disorders were included in two of the trials (Canuso et al. 2010a; Canuso et al. 2010b). The dosing regimen used in these trials was flexible. Other PAL-ER trials and all PP trials used a fixed dosing regimen.

Table S3. Transitivity assessment.

	Boxplot	Kruskal–Wallis equality of populations rank test
Total number of participant	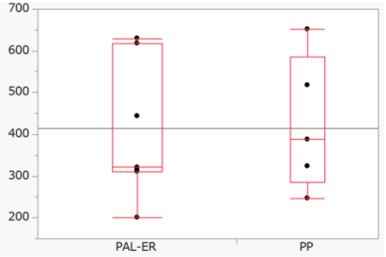	Chi-squared with ties = 0.3231 (df = 1), p = 0.5698
Proportion of males (%)	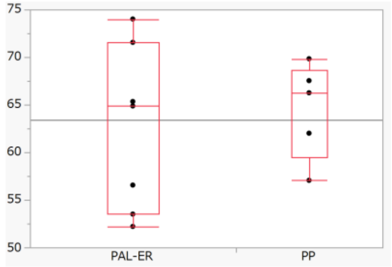	Chi-squared with ties = 0.3231 (df = 1), p = 0.5698
Age (years)	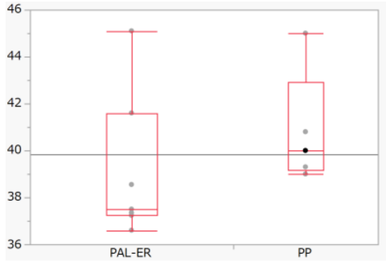	Chi-squared with ties = 1.906 (df = 1), p = 0.1675
PANSS-T score at baseline	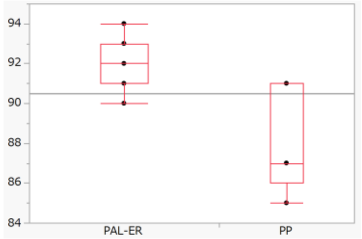	Chi-squared with ties = 5.684 (df = 1), p = 0.0171

Table S4. The results of network meta-analysis

Outcome	Between study variance	Heterogeneity assessment	CINeMA confidence rating for PP vs PAL-ER*
PANSS total scores at 6 weeks	7.710	Low	Very low
Discontinuation due to inefficacy	0.032	Low to moderate	Very low
All-cause discontinuation	0.019	Low	Very low
Discontinuation due to adverse event	0.000	Low	Very low
Discontinuation due to withdrawal of consent	0.000	Low	Very low
Agitation	0.000	Low	Very low
Use of anticholinergic agents	0.070	Low to moderate	Very low
Akathisia	0.110	Moderate to high	Very low
Extrapyramidal symptoms	0.086	Low to moderate	Very low
Somnolence	0.273	High	Very low
Dizziness	0.167	High	Very low
Insomnia	0.007	Low	Very low
Headache	0.099	Moderate to high	Very low
Constipation	0.222	High	Very low
Nausea	0.032	Low to moderate	Very low
Vomiting	0.224	High	Very low
Weight gain	0.000	Low	Very low
Weight change	0.204	Low	Very low
Blood prolactin change in male	0.475	Low	Very low
Blood prolactin change in female	100.202	High	Very low

Change in PANSS total scores: differences outside the interval -3 to 3 points are considered clinically important. For dichotomous outcomes, the clinically meaningful threshold was set at an odds ratio of 0.8 and 1.25 for comparisons of two antipsychotics. Change in body weight: differences outside the interval -2 kg to +2 kg are considered clinically important. Change in prolactin: differences outside the interval -10 ug/l to +10 ug/mL are considered clinically important.

References

Canuso CM, Lindenmayer JP, Kosik-Gonzalez C, Turkoz I, Carothers J, Bossie CA, Schooler NR (2010a) A randomized, double-blind, placebo-controlled study of 2 dose ranges of paliperidone extended-release in the treatment of subjects with schizoaffective disorder. J Clin Psychiatry 71: 587-98.

Canuso CM, Schooler N, Carothers J, Turkoz I, Kosik-Gonzalez C, Bossie CA, Walling D, Lindenmayer JP (2010b) Paliperidone extended-release in schizoaffective disorder: a randomized, controlled study comparing a flexible dose with placebo in patients treated with and without antidepressants and/or mood stabilizers. J Clin Psychopharmacol 30: 487-95.

Coppola D, Melkote R, Lannie C, Singh J, Nuamah I, Gopal S, Hough D, Palumbo J (2011) Efficacy and Safety of Paliperidone Extended Release 1.5 mg/day-A Double-blind, Placebo- and Active-Controlled, Study in the Treatment of Patients with Schizophrenia. Psychopharmacol Bull 44: 54-72.

Davidson M, Emsley R, Kramer M, Ford L, Pan G, Lim P, Eerdekens M (2007) Efficacy, safety and early response of paliperidone extended-release tablets (paliperidone ER): results of a 6-week, randomized, placebo-controlled study. Schizophr Res 93: 117-30.

Gopal S, Hough DW, Xu H, Lull JM, Gassmann-Mayer C, Remmerie BM, Eerdekens MH, Brown DW (2010) Efficacy and safety of paliperidone palmitate in adult patients with acutely symptomatic schizophrenia: a randomized, double-blind, placebo-controlled, dose-response study. Int Clin Psychopharmacol 25: 247-56.

Hirayasu Y, Tomioka M, Iizumi M, Kikuchi H (2010) A double-blind, placebo-controlled, comparative study of paliperidone Extended-Release (ER) tablets in patients with schizophrenia. Jpn J Clin Psychopharmacol 13: 2077-2103.

Kane J, Canas F, Kramer M, Ford L, Gassmann-Mayer C, Lim P, Eerdekens M (2007) Treatment of schizophrenia with paliperidone extended-release tablets: a 6-week placebo-controlled trial. Schizophr Res 90: 147-61.

Kramer M, Litman R, Hough D, Lane R, Lim P, Liu Y, Eerdekens M (2010) Paliperidone palmitate, a potential long-acting treatment for patients with schizophrenia. Results of a randomized, double-blind, placebo-controlled efficacy and safety study. Int J Neuropsychopharmacol 13: 635-47.

Marder SR, Kramer M, Ford L, Eerdekens E, Lim P, Eerdekens M, Lowy A (2007) Efficacy and safety of paliperidone extended-release tablets: results of a 6-week, randomized, placebo-controlled study. Biol Psychiatry 62: 1363-70.

Nasrallah HA, Gopal S, Gassmann-Mayer C, Quiroz JA, Lim P, Eerdekens M, Yuen E, Hough D (2010) A controlled, evidence-based trial of paliperidone palmitate, a long-acting injectable antipsychotic, in schizophrenia. Neuropsychopharmacology 35: 2072-82.

Pandina GJ, Lindenmayer JP, Lull J, Lim P, Gopal S, Herben V, Kusumakar V, Yuen E, Palumbo J (2010) A randomized, placebo-controlled study to assess the efficacy and safety of 3 doses of paliperidone palmitate in adults with acutely exacerbated schizophrenia. J Clin Psychopharmacol 30: 235-44.

Takahashi N, Takahashi M, Saito T, Iizumi M, Saito Y, Shimizu H, Matsumura T (2013) Randomized, placebo-controlled, double-blind study assessing the efficacy and safety of paliperidone palmitate in Asian patients with schizophrenia. Neuropsychiatr Dis Treat 9: 1889-98.
